# Interregional mobility in different age groups is associated with COVID-19 transmission in the Taipei metropolitan area, Taiwan

**DOI:** 10.1038/s41598-023-44474-z

**Published:** 2023-10-12

**Authors:** Wei-Ming Jiang, Tzai-Hung Wen, Ying-Chi Huang, Hung-Yi Chiou, Wei J. Chen, Chao A. Hsiung, Huey-Kang Sytwu, Hsiao-Hui Tsou

**Affiliations:** 1 Institute of Population Health Sciences, National Health Research Institutes, 35 Keyan Road, Zhunan, 350 Miaoli County Taiwan; 2 Department of Geography, National Taiwan University, Taipei, Taiwan; 3 National Institute of Infectious Diseases and Vaccinology, National Health Research Institutes, Zhunan, Miaoli County Taiwan; 4 School of Public Health, College of Public Health, Taipei Medical University, Taipei, Taiwan; 5 Master’s Program in Applied Epidemiology, College of Public Health, Taipei Medical University, Taipei, Taiwan; 6 Center for Neuropsychiatric Research, National Health Research Institutes, Zhunan, Miaoli County Taiwan; 7 Institute of Epidemiology and Preventive Medicine, College of Public Health, National Taiwan University, Taipei, Taiwan; 8 Graduate Institute of Biostatistics, College of Public Health, China Medical University, Taichung, Taiwan

**Keywords:** Infectious diseases, Viral infection, Computational biology and bioinformatics, Computational models

## Abstract

Before vaccines were introduced, mobility restriction was one of the primary control measures in the early stage of the coronavirus disease 2019 (COVID-19) pandemic. Because different age groups face disproportionate health risks, differences in their mobility changes affect the effectiveness of pandemic control measures. This study aimed to investigate the relationship between multiscale mobility patterns in different age groups and COVID-19 transmission before and after control measures implementation. Data on daily confirmed case numbers, anonymized mobile phone data, and 38 socioeconomic factors were used to construct negative binomial regression models of these relationships in the Taipei metropolitan area in May 2021. To avoid overfitting, the socioeconomic factor dimensions were reduced by principal component analysis. The results showed that inter-district mobility was a greater promoter of COVID-19 transmission than was intra-district mobility (coefficients: pre-alert, 0.52 and 0.43; post-alert, 0.41 and 0.36, respectively). Moreover, both the inter-district mobility of people aged 15–59 and ≥ 60 years were significantly related to the number of confirmed cases (coefficients: pre-alert, 0.82 and 1.05; post-alert, 0.48 and 0.66, respectively). The results can help agencies worldwide formulate public health responses to emerging infectious diseases.

## Introduction

From the end of December 2019, severe acute respiratory syndrome coronavirus 2 (SARS-CoV-2) caused a coronavirus disease 2019 (COVID-19) pandemic worldwide. By November 2022, more than 632 million confirmed COVID-19 cases and more than 6 million deaths with COVID-19 had been reported across 193 countries and regions^1^. Because COVID-19 is a respiratory disease, most countries quickly implemented control measures including mobility restrictions (e.g., travel) and non-pharmaceutical interventions (NPIs; e.g., social distancing, face mask use) to mitigate outbreaks before vaccines were distributed widely. Before the implementation of mobility restrictions, COVID-19 should spread relatively randomly and be uniformly distributed in population flows due to a relative lack of awareness^2^. Because human mobility can result in early shifts of the epidemiological dynamics from epidemics driven by frequent importation into community transmission^3^, human mobility played an important role in COVID-19 transmission^4,5^.

Several studies have analyzed the relationship between human mobility and COVID-19 transmission by spatial–temporal mobility data from various sources^4,5^. These studies mainly relied on three types of mobility data. The first type of mobility data is public transit systems such as buses, trains, metro, ferries, and air flights. The mobility data including service capability, travel time, trip information, and boarding and alighting locations can be retrieved from scheduled timetable data and actual travel records of passengers^6–8^. However, this type of mobility data is only able to cover the mobility changes between boarding and alighting locations in public transit systems. The second type of mobility data is social media mobile apps such as Facebook, Twitter, Google Mobility Report, and Baidu Mobility Index^3,9–13^. Social media mobile apps can offer aggregated and anonymized mobility data from users who have turned on the global positioning system (GPS) or posted content with geotagging. However, the limitations of each social media mobile app are different. For example, Baidu Mobility Index only contains mobility data in Mainland China. Although Google Mobility Report can provide mobility changes to the point of interest such as residential areas, workplaces, and transit stations, it cannot estimate inter-regional mobility. The third type of mobility data is mobile network operators. Mobile network operators decode signaling information between opening mobile phones and cell sites to provide anonymized and aggregated mobility data. The advantage of mobile phone data is that it is not limited to the analysis of mobility changes in boarding and alighting stations or particular locations^14^. Mobile phone data can offer extensive and representative mobility information at the population level to investigate intra- and inter-regional mobility changes between origin–destination geographic scales such as city and county^2,15–17^.

According to the origin and destination locations of human mobility, mobility patterns can be classified into two types: intra-regional flows and inter-regional flows^18,19^. The intra-regional flows are mainly responsible for local diffusion within specific areas such as workplaces and residential locations. For example, Nagata et al. (2021) found that the mobility changes at nightlife locations were related more significantly to COVID-19 outbreaks than were changes at workplaces and residential locations^20^. On the other hand, the inter-regional flows contribute to the spatial spread of the epidemic from outbreak locales to other unaffected or weakly affected areas. For example, Jia et al. (2020) predicted the geographical distribution of infections according to population outflows from Wuhan to other provinces in China between 1 and 24 January 2020^2^. Because the intra- and inter-regional flows play different roles in the spread of the epidemic, evaluating their impacts on transmission can help agencies formulate more specific prevention policies to mitigate COVID-19 outbreaks.

Given the age-dependent effects on COVID-19 transmission and health risk, researchers have investigated mobility changes in different age groups after control measures implementation^21–23^. For instance, using anonymized and aggregated mobility indicators, Caselli et al. (2022) found that the mobility of people aged 18–24 years in Italy, Portugal, and Spain declined by > 20% after the 2020 introduction of stay-at-home orders, whereas that of people aged ≥ 65 years declined by only 11%, representing a significant difference^22^. To prevent bias and the mismeasurement of control measure effects due to the aggregation of data from all age groups, the heterogeneous responses of people in different age groups to control measures need to be clarified. However, there is still a lack of research to compare the impacts of intra- and inter-regional flows in different age groups on COVID-19 transmission.

This study aims to investigate the relationship between inter- and intra-regional population flows in different age groups and COVID-19 transmission before and after control measures implementation. There are two research questions. First, which one of inter- and intra-regional flows was related more significantly to COVID-19 transmission? Second, whether there was a significantly different relationship between mobility patterns in different age groups and COVID-19 transmission? To answer these two research questions, the study conducted negative binomial regression models to analyze the relationship between mobility patterns in different age groups and the number of confirmed COVID-19 cases before and after control measures implementation. In addition, socioeconomic factors are the important determinants of COVID-19 infection^6,24,25^. Considering the influence of socioeconomic factors on the relationship between human mobility and COVID-19 transmissions, this study included 38 socioeconomic factors as covariates in regression models. To avoid overfitting and collinearity, socioeconomic factor dimensions were reduced by principal component analysis.

Due to the implementation of a strict quarantine policy, ≥ 90% of COVID-19 cases recorded in Taiwan through April 2021 were imported from other countries^26^. In May 2021, the B.1.1.7 variant of SARS-CoV-2 caused the first local outbreak in the Taipei metropolitan area. To control it, the National Health Command Center declared a level-3 COVID-19 alert in this area on 15 May 2021. All schools were closed, with teaching conducted online; members of the public were advised to avoid unnecessary travel; face mask use outside of the home was required at all times; indoor gatherings of > 5 people and outdoor gatherings of > 10 people were prohibited. Because this wave of the epidemic was independent and the timing of policy intervention is clear, we selected this context to compare the relationships between mobility patterns, overall and by age group, and COVID-19 transmission before and after control measures implementation.

### Study area

**Figure 1:**
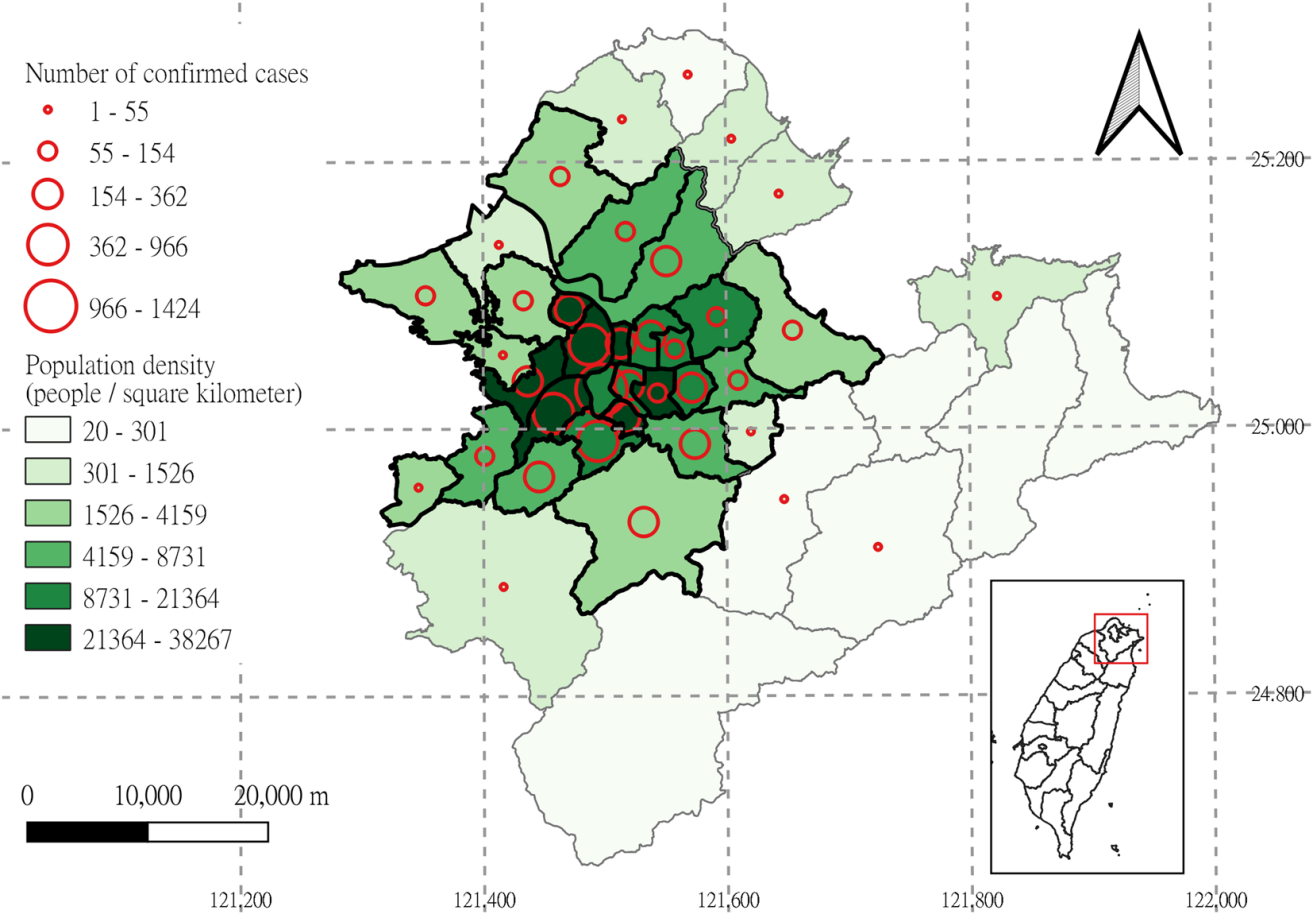
Study area. The study area comprises 29 districts in the Taipei metropolitan area (black outline). The numbers of confirmed COVID-19 cases were accumulated from 3 to 31 May 2021.

The study area comprises 29 districts in the Taipei metropolitan area, which consists of Taipei City and New Taipei City and is the most densely populated region in Taiwan (Fig. 1). Most confirmed COVID-19 cases reported between 3 and 31 May 2021 were in urban areas. According to the definition of the Department of Statistics of Taiwan’s Ministry of the Interior, this study selected 29 districts, with population densities ≥ 1000 people/km^2^, as the study area^27^. In Taiwan, districts are administrative subdivisions of special municipalities and provincial cities. Although most people’s daily activities occur in their districts of residence, > 68% of people in the Taipei metropolitan area commute across districts daily^28^.

## Methods

### Research designs

**Figure 2:**
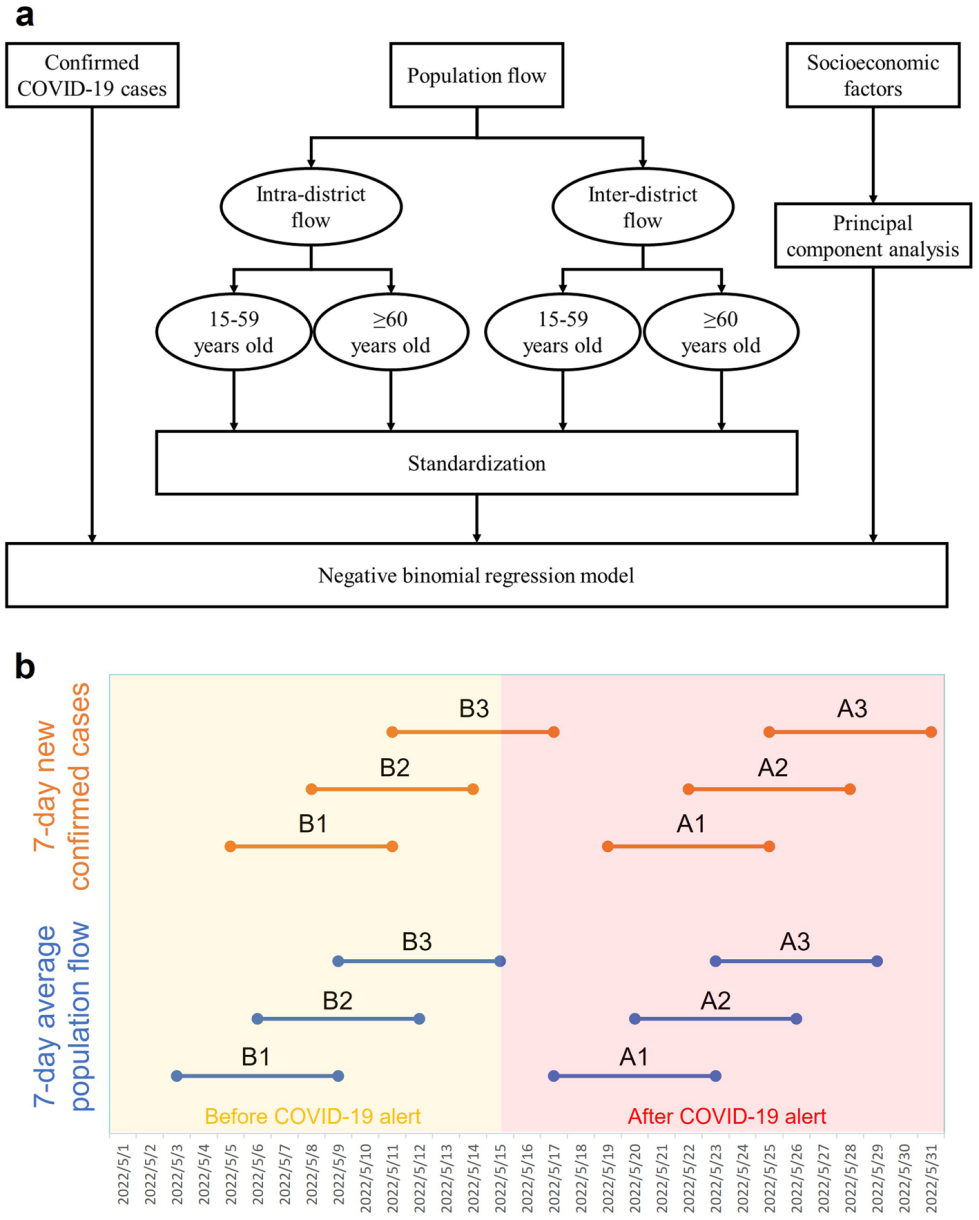
Flow chart and model structure. The flow chart of the overall analysis (**a**) and the model structure of regression models (**b**). B1 to B3 and A1 to A3 represent the three selected periods before and after the COVID-19 alert implementation, respectively.

Figure 2a shows the flow chart of the overall analysis in this study. The dependent variable and the independent variable were the numbers of confirmed COVID-19 cases and population flows, respectively. Socioeconomic factors were included as covariates. There were three steps for the preprocessing of population flows. First, the population flows were classified into two categories: intra-district flows and inter-district flows. Second, the population flows were sorted into people aged 15–59 years and people ≥ 60 years by age groups. Third, the population flows were standardized by means and standard deviations to have unit variance which is beneficial to compare regression coefficients. On the other hand, this study conducted the principal component analysis to obtain principal components of the socioeconomic factors. To compare the changes in the relationship between human mobility and COVID-19 transmission, the study selected three periods before and after the COVID-19 alert implementation on 15 May 2021 (Fig. 2b). Because of the COVID-19 incubation period and reporting delay, the population flows a few days previously led to the number of new confirmed COVID-19 cases in each period.

### Data

**Figure 3:**
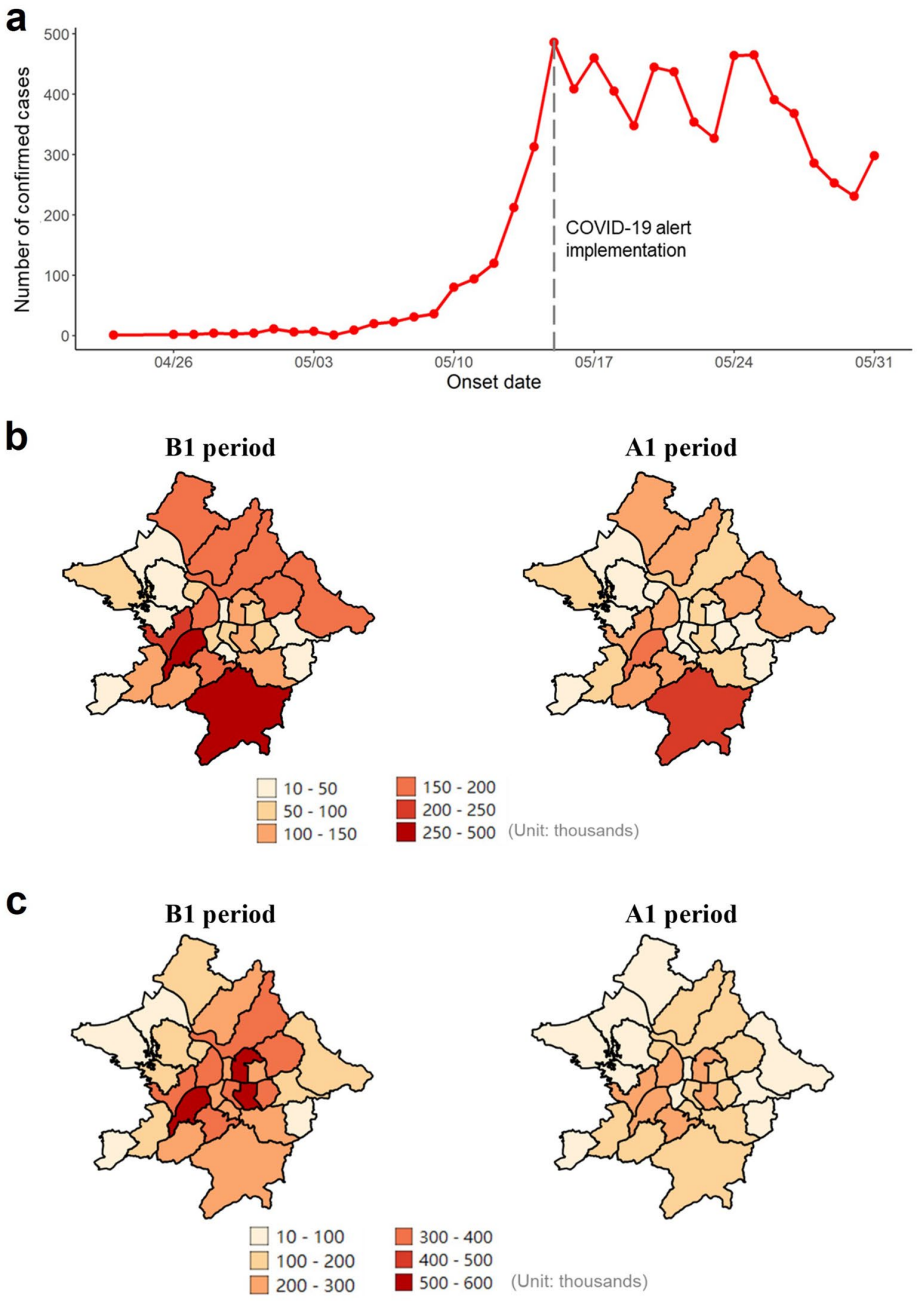
Change in the number of confirmed COVID-19 cases and different mobility patterns. The daily number of confirmed new COVID-19 cases (**a**), 7-day average of the total number of district-level intraflows (**b**), and interflows (**c**) following level-3 COVID-19 alert. B1 period, 3–9 May 2021; A1 period, 17–23 May 2021.

Daily district-level data on the numbers of confirmed COVID-19 cases in Taiwan were obtained from the Taiwan Centers for Disease Control’s open data portal^29^. Data on local (i.e., not imported) cases occurring in the study area between 5 and 31 May 2021 were extracted, according to patients’ districts of residence and self-reported onset dates. During this period, COVID-19 cases were confirmed by the polymerase chain reaction. In the study area, the daily number of new confirmed COVID-19 cases increased steeply at the beginning of the outbreak, peaked on May 15, and then fluctuated dramatically between 16 and 31 May (Fig. 3a).

Population flow was examined using daily mobile phone data obtained from Far EasTone Telecommunications, a Taiwan-based company that held roughly 24.6% of the national market share in 2019^30^. The company decoded information on signals between active mobile phones and cell sites to provide anonymized and aggregated mobility data (millions of data points) for the study area and period. The number of trips was weighted by poststratification using national census data^31^. Therefore, the mobile phone data were considered to comprise a representative sample of the population flow structure in the study area. This mobile phone data is origin–destination data which is measured as the total number of trips from origin to destination per day. The spatial resolution of the original mobile phone data was at the village level. According to the origin and destination villages, population flow was aggregated at the district level and classified as intra-district flows and inter-district flows. Intra- and inter-district flows decreased visually following control measure implementation on 15 May (Fig. 3b,c). Population flow was also determined separately for people aged 15–59 and ≥ 60 years. To eliminate the effects of daily fluctuations, standardized 7-day average population flows were calculated.

District-level data on 38 socioeconomic (demographic, education, economic, healthcare, equipment modernity, and land use) factors were collected from the government’s open data platforms^27,32,33^ (Supplementary Table S1). Data pertaining to months as close as possible to May 2021 were extracted.

### Statistical analysis

Because the number of socioeconomic variables was too large for simultaneous model fitting, principal component analysis was conducted to resolve model overfitting and/or collinearity^6,34^. If most of the total population variance could be attributed to the first one, two, or three principal components (PCs), those components could replace the original variables without much loss of information. Because the socioeconomic variables were measured at widely differing scales, they were standardized to avoid the domination of loadings by specific variables. PCs were obtained from the standardized variables using the following formula^35^.

$$Z_{i} = {\varvec{e}}_{i}^{\prime} \left( {{\varvec{X}} - {\varvec{\mu}}} \right)\left( {{\varvec{V}}^{1/2} } \right)^{ - 1} , \quad i = 1, 2, \cdots , p,$$

where ${Z}_{i}$ represents the PCs, ${{\varvec{e}}}_{i}{\prime}$ represents the transpose of eigenvectors, ${\varvec{X}}$ represents the socioeconomic variables, ${\varvec{\mu}}$ represents the means of the socioeconomic variables, and ${\left({{\varvec{V}}}^{1/2}\right)}^{-1}$ represents the inverse of diagonal standard deviation matrices.

Because confirmed case variance is often greater than the mean, referred to as overdispersion^8,36^, the data were analyzed using negative binomial regression models. These models can be used to analyze strictly positive response variables, such as counts and rates, and variable variance and means do not need to be equal. In these models, the dependent and independent variables were the numbers of confirmed COVID-19 cases and population flow, respectively, and the socioeconomic factors were included as covariates. Coefficients obtained before and after level-3 COVID-19 alert implementation on 15 May 2021 were compared. Negative binomial regression was conducted using the MASS package in R (version 3.6.3) and the following formula^37,38^:

$$\ln {\text{E}}\left( {y_{i} } \right) = \alpha + \beta_{1} \cdot \mathop \sum \limits_{j = 1, j \ne i}^{m} x_{ji} + \beta_{2} \cdot x_{ii} + \mathop \sum \limits_{k = 1}^{n} \gamma_{k} \cdot z_{k} ,$$

where $\mathrm{\rm E}\left({y}_{i}\right)$ represents the expected number of confirmed COVID-19 cases in $i$ district; $\alpha$ represents the intercept; ${x}_{ji}$ represents the standardized 7-day average inflow from district $j$ to district $i$; m represents the number of districts in the study area; ${x}_{ii}$ represents the standardized 7-day average intra-district flow within district $i$; ${z}_{k}$ represents the PCs of the socioeconomic factors; $n$ represents the number of PCs selected; and ${\beta }_{1}$, ${\beta }_{2}$, and ${\gamma }_{k}$ are regression coefficients.

More specifically, the following formula was conducted when the inter- and intra-flows were separated into people aged 15–59 years and people ≥ 60 years by age groups.

$$\begin{aligned} \ln {\text{E}}\left( {y_{i} } \right) & = \alpha + \beta_{1}^{Age15 - 59} \cdot \mathop \sum \limits_{j = 1, j \ne i}^{m} x_{ji}^{Age15 - 59} + \beta_{1}^{Age \ge 60} \cdot \mathop \sum \limits_{j = 1, j \ne i}^{m} x_{ji}^{Age \ge 60} \\ & \quad +\, \beta_{2}^{Age15 - 59} \cdot x_{ii}^{Age15 - 59} + \beta_{2}^{Age \ge 60} \cdot x_{ii}^{Age \ge 60} + \mathop \sum \limits_{k = 1}^{n} \gamma_{k} \cdot z_{k} , \\ \end{aligned}$$

where ${x}_{ji}^{Age15-59}$ and ${x}_{ji}^{Age\ge 60}$ represents the standardized 7-day average inflows of people aged 15–59 years and people ≥ 60 years from district $j$ to district $i$, respectively; ${x}_{ii}^{Age15-59}$ and ${x}_{ii}^{Age\ge 60}$ represents the standardized 7-day average intra-district flows of people aged 15–59 years and people ≥ 60 years within district $i$, respectively; and ${\beta }_{1}^{Age15-59}$, ${\beta }_{1}^{Age\ge 60}$, ${\beta }_{2}^{Age15-59}$, and ${\beta }_{2}^{Age\ge 60}$ are corresponding regression coefficients.

The maps were visualized using free and open geographic information system software QGIS (version 3.14), and the charts were plotted via the ggplot2 package (version 3.3.3) in R and Microsoft Excel (version 2210)^39–41^.

### Ethical approval

The institutional review board (IRB) of the National Health Research Institutes approved this study (EC1091110-E-R1). This study does not involve live animals and individual human participants. The requirement for informed consent from the study subjects was waived by the IRB of the National Health Research Institutes due to the reason that the mobile phone data analyzed in this study are de-identification, anonymized, and aggregated on a district level. No personally identifiable information, such as an individual’s location, contacts, or movement, was made available at any point. This study confirms that all methods were performed in accordance with the relevant guidelines and regulations.

## Results

### Principal component analysis results

**Figure 4:**
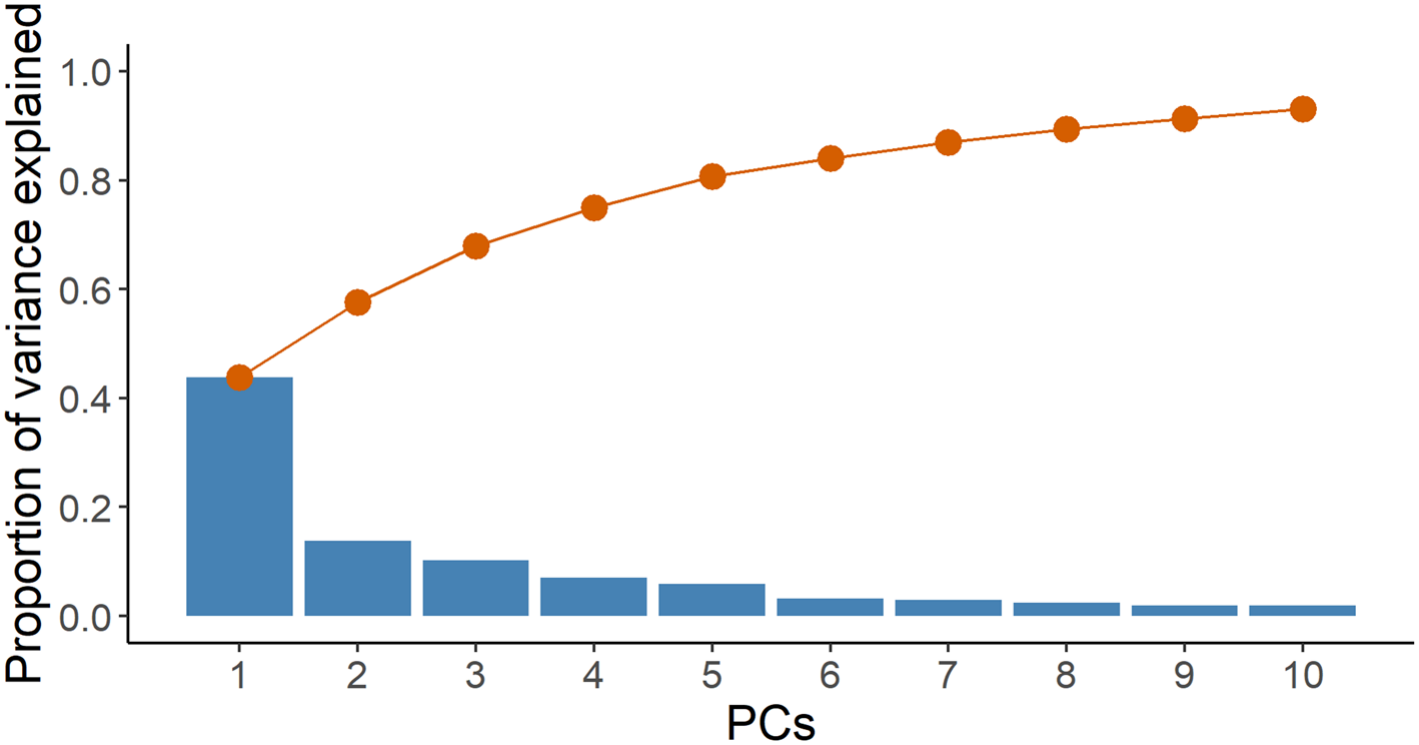
The proportion of variance explained by the first 10 PCs. The blue bars indicate the proportion of variance explained by PCs. The red lines represent the cumulative proportion of total variance.

Figure 4 shows the proportion of variance explained by the first 10 PCs. Because the first 3 PCs explained roughly 70% of the total sample variance, they served as covariates in the models. PC1, PC2, and PC3 represented mainly the education level, demographic structure, and combined equipment modernity and economic development, respectively (Supplementary Table S2). PC1 was dominated by the percentages of the population that had completed senior high school (variable loading = 0.23) and obtained master’s degrees (variable loading = − 0.24). PC2 was dominated by the aging index (variable loading = 0.37) and natural increase rate (variable loading = − 0.39). PC3 was dominated by the percentage of middle-low-income families (variable loading = 0.34) and the popularity of cable television (variable loading = − 0.32).

### Relationships among mobility patterns, PCs, and confirmed case numbers

**Figure 5:**
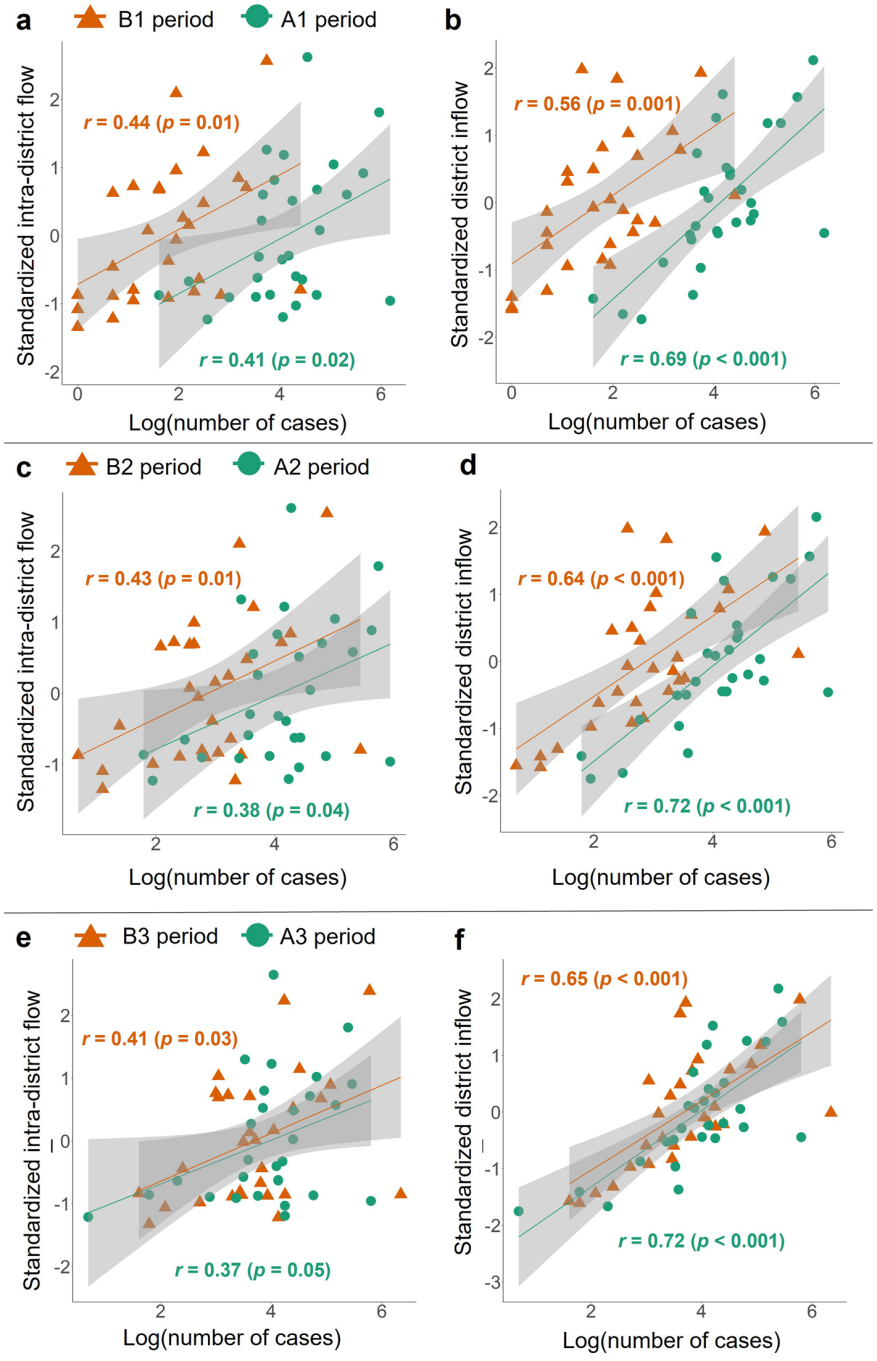
Pearson correlations among the number of confirmed COVID-19 cases, intra-district flow, and district inflow. The correlations among the natural logarithm of the number of confirmed COVID-19 cases per district, standardized intra-district flow (**a**, **c**, **e**), and standardized district inflow (**b**, **d**, **f**) before (B1, 2, 3 periods) and after (A1, 2, 3 periods) COVID-19 alert implementation. The regression lines were fitted with linear models. Gray shading represents 95% confidence intervals.

Pearson coefficients of correlation between the logarithm of the number of confirmed cases and standardized intra-district flows in periods B1 and A1 were close to 0.4, and the relationship between these variables was linear and positive (Fig. 5a). The coefficients of correlation between the logarithm of the number of confirmed cases and standardized district inflows in periods B1 and A1 were 0.56 and 0.69, respectively, with a positive linear relationship (Fig. 5b). In addition, all relationships between the logarithm of the number of confirmed cases and standardized intra-district flows and district inflows in periods B2,3 and A2,3 were positive and linear (Fig. 5c,d,e,f).

The sum of intra-district flow and district inflow was associated positively with the number of confirmed cases, with coefficients 0.6, 0.59, and 0.52 in periods B1,2,3 and 0.44, 0.45, and 0.42 in periods A1,2,3 and no significant difference between periods (Supplementary Table S3a, 4). At the district level, coefficients for intra-district flow were not significant in either period, whereas those for inflow were positive and significant (coefficients, 0.81, 0.81, 0.78 in periods B1,2,3; 0.59, 0.61, 0.59 in periods A1,2,3), with no significant difference between periods (Supplementary Table S3b,c, 5). The number of confirmed cases was associated more strongly with inflow (coefficients, 0.92, 0.92, 0.95 in periods B1,2,3; 0.65, 0.69, 0.66 in periods A1,2,3) than with intra-district flow (Supplementary Table S3d). No collinearity between intra-district flow and inflow was detected in any of the six periods (variance inflation factors < 5, *r* = 0.45–0.50; Supplementary Table S6).

In the model including the PCs, the sum of intra-district flows and district inflow was associated positively with the number of confirmed COVID-19 cases (coefficients: 0.84, 0.83, 0.81 in periods B1,2,3; 0.59, 0.68, 0.66 in periods A1,2,3; Table 1a, Supplementary Table S7a). The exponentiated coefficient, such as (${e}^{0.84}$) in period B1, is the incidence rate ratio (IRR). In periods B1 and A1, the number of confirmed cases increased by a factor of 2.32 (${e}^{0.84}$) and 1.80 (${e}^{0.59}$) times, respectively, for every unit increase in mobility. The number of confirmed cases was related more strongly to PC2 and PC3 than to PC1.

**Table 1:** Regression associations among mobility patterns, PCs, and the number of confirmed COVID-19 cases.

		B1 period		A1 period	
		Coefficient (std. error)	*p*	Coefficient (std. error)	*p*
a	Intercept	1.80 (0.15)	< 0.001***	4.32 (0.12)	< 0.001***
	Intraflow + Interflow	0.84 (0.18)	< 0.001***	0.59 (0.14)	< 0.001***
	PC1	0.07 (0.04)	0.12	− 0.009 (0.03)	0.77
	PC2	0.24 (0.07)	0.001**	0.05 (0.06)	0.36
	PC3	0.20 (0.08)	0.01*	0.13 (0.07)	0.06
	Pseudo *r*^2^	0.61		0.55	
b	Intercept	1.84 (0.16)	< 0.001***	4.32 (0.12)	< 0.001***
	Intraflow	0.79 (0.19)	< 0.001***	0.73 (0.15)	< 0.001***
	PC1	− 0.03 (0.04)	0.48	− 0.07 (0.03)	0.03*
	PC2	0.26 (0.08)	0.001**	0.17 (0.05)	0.002**
	PC3	0.30 (0.10)	0.002**	0.23 (0.08)	0.002**
	Pseudo *r*^2^	0.57		0.57	
c	Intercept	1.82 (0.16)	< 0.001***	4.41 (0.14)	< 0.001***
	Interflow	0.89 (0.21)	< 0.001***	0.49 (0.17)	0.003**
	PC1	0.11 (0.05)	0.03*	0.02 (0.04)	0.61
	PC2	0.23 (0.08)	0.003**	0.01 (0.06)	0.85
	PC3	0.12 (0.08)	0.14	0.03 (0.07)	0.67
	Pseudo *r*^2^	0.57		0.39	
d	Intercept	1.80 (0.15)	< 0.001	4.28 (0.11)	< 0.001***
	Intraflow	0.43 (0.24)	0.07	0.36 (0.17)	0.04*
	Interflow	0.52 (0.27)	0.05	0.41 (0.17)	0.01*
	PC1	0.05 (0.06)	0.37	− 0.01 (0.04)	0.70
	PC2	0.24 (0.07)	0.001**	0.11 (0.05)	0.03*
	PC3	0.23 (0.10)	0.02*	0.16 (0.07)	0.03*
	Pseudo *r*^2^	0.61		0.64	

*p < 0.05, **p < 0.01, ***p < 0.001.

In the model including the PCs, intra-district flow was associated positively with the number of confirmed COVID-19 cases pre- and post-alert, with coefficients 0.79, 0.78, 0.79 in periods B1,2,3 and 0.73, 0.73, 0.72 in periods A1,2,3 (Table 1b, Supplementary Table S7b). In periods B1 and A1, the number of confirmed cases increased by a factor of 2.20 (${e}^{0.79}$) and 2.08 (${e}^{0.73}$) times, respectively, for every unit increase in intra-district flow. The coefficients for PC2 and PC3 were positive and significant before and after alert implementation, whereas those for PC1 were negative and only significant post-alert.

Inflow was also associated significantly with the number of confirmed COVID-19 cases in the model including the PCs, with coefficients 0.89, 0.87, 0.84 in periods B1,2,3 and 0.49, 0.64, 0.60 in periods A1,2,3 (Table 1c, Supplementary Table S7c). The number of confirmed cases increased by a factor of 2.44 $$({e}^{0.89}$$) times for every unit increase in inflow in period B1 and by a factor of 1.63 ($${e}^{0.49})$$ times in period A1. The coefficients for PC2 were positive and significant before and after alert implementation, except in the A1 period, and those for PC1 were significant only before alert implementation.

In the model including the PCs, the number of confirmed COVID-19 cases was related positively to intra-district flow and district inflow, except in the B1 period (Table 1d, Supplementary Table S7d). Pre- and post-alert, this relationship was stronger for district inflow (coefficients, 0.52, 0.48, 0.48 in periods B1,2,3 and 0.41, 0.46, 0.43 in periods A1,2,3) than for intra-district flow (coefficients, 0.43, 0.45, 0.45 in periods B1,2,3 and 0.36, 0.33, 0.35 in periods A1,2,3). PC2 and PC3 were related more strongly than PC1 to the number of confirmed cases.

### Relationships among mobility patterns in different age groups, PCs, and confirmed case numbers

**Figure 6:**
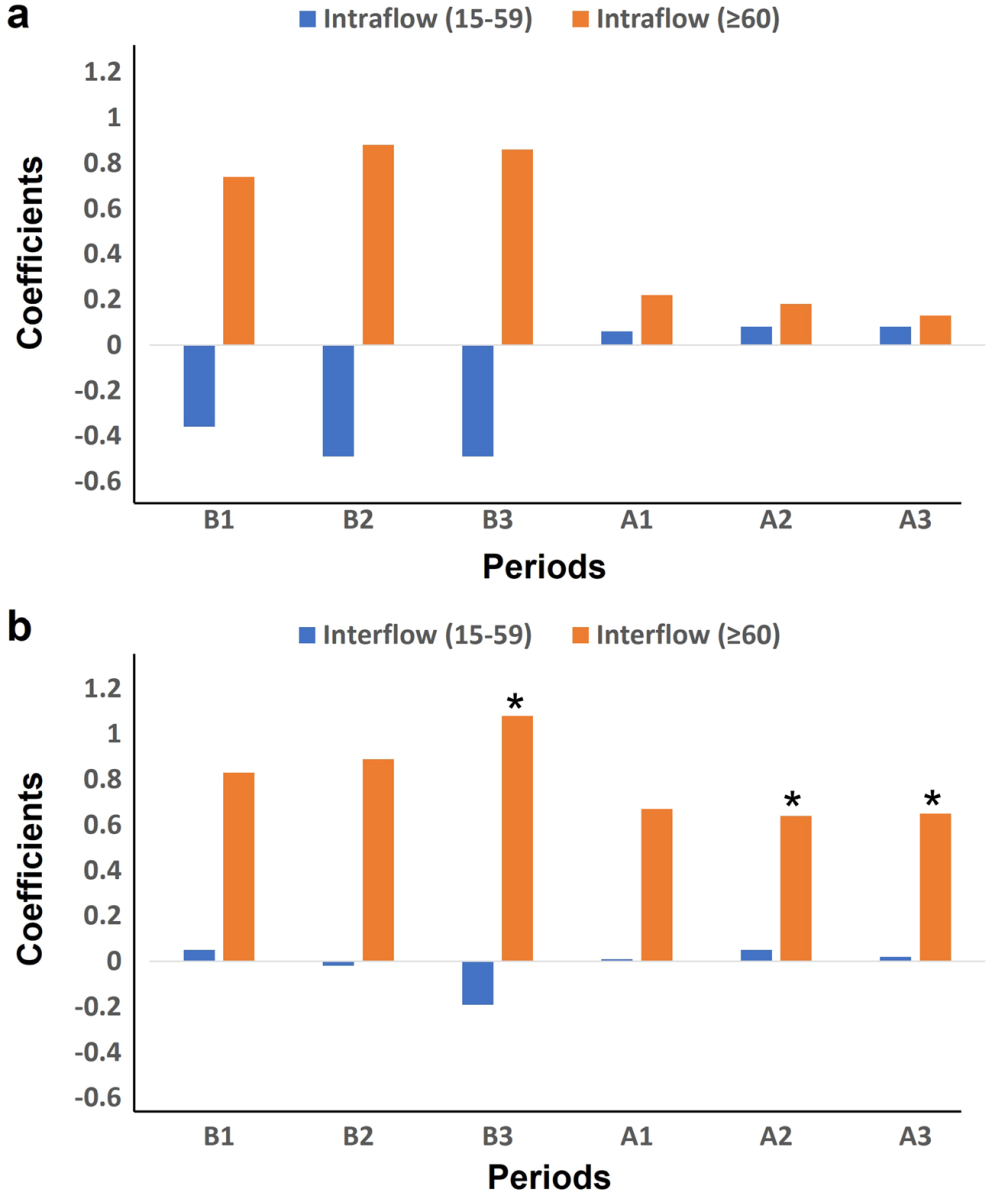
Regression coefficients between the number of confirmed COVID-19 cases and mobility patterns in different age groups. The regression coefficients between the intra- and inter-district flows (a, b) of people aged 15–59 and ≥ 60 years and the number of confirmed COVID-19 cases. *p < 0.05.

The intra-district flows of people aged 15–59 years were related significantly to the number of confirmed COVID-19 cases before and after alert implementation (coefficients, 0.80, 0.79, 0.80 in periods B1,2,3; 0.75, 0.76, 0.76 in periods A1,2,3; Supplementary Table S8a). In addition, there was also a significant relationship between the intra-district flows of people aged ≥ 60 years and the number of confirmed COVID-19 cases before and after alert implementation (coefficients, 0.76, 0.77, 0.78 in periods B1,2,3; 0.67, 0.64, 0.61 in periods A1,2,3; Supplementary Table S8b). However, as shown in Fig. 6a based on Supplementary Table S8c, no such relationship was observed when both age groups were considered simultaneously. This is probably due to the issue of multicollinearity between the intra-district flows of people aged 15–59 and ≥ 60 years (Supplementary Table S10).

In the model including the PCs, the inflows of people aged 15–59 years were related significantly to the number of confirmed cases (coefficients: 0.82, 0.81, 0.78 in periods B1,2,3; 0.48, 0.60, 0.57 in periods A1,2,3; Supplementary Table S9a). Furthermore, the inflows of people aged ≥ 60 years were also related significantly to the number of confirmed cases (coefficients: 1.05, 1.01, 0.99 in periods B1,2,3; 0.66, 0.74, 0.70 in periods A1,2,3; Supplementary Table S9b).

However, as shown in Fig. 6b based on Supplementary Table S9c, when the inflows of people aged 15–59 and ≥ 60 years were considered simultaneously, the inflows of people aged 15–59 and ≥ 60 years showed no significant relationship, even though the inflows of people aged ≥ 60 years were weakly significant (*p*-values ranging from 0.01 to 0.05) in periods B3 and A2,3. This is probably because the results suffer from an issue on the multicollinearity, there are high correlations between the inflows of people aged 15–59 and ≥ 60 years (Supplementary Table S11).

## Discussion

The purpose of this study was to examine the relationship between mobility patterns in different age groups and COVID-19 transmission in the Taipei metropolitan area before and after the implementation of control measures. It showed that intra-district flow and district inflow were related positively to the number of confirmed COVID-19 cases before and after alert implementation, with the latter relationship stronger than the former and attenuating markedly post-alert. Moreover, both the inter-district flows of people aged 15–59 and ≥ 60 years were significantly related to the number of confirmed cases. Lastly, PC2 and PC3 were related positively to the number of confirmed COVID-19 cases before and after alert implementation.

The finding of the relationship between district inflow and COVID-19 transmission in this study may reflect the mobility behaviors in the Taipei metropolitan area. There is a tendency for people to congregate in districts with high population densities and a high percentage of cross-district mobility in the Taipei metropolitan area^28^. On the other hand, the intra-district flow commonly represents the daily activities in neighborhoods such as grocery shopping, and it is less affected by travel restrictions. This finding is consistent with those of Jia et al. (2020) and Wei and Wang (2020), who observed that movement out of the COVID-19 outbreak center of Wuhan was related significantly to the number of confirmed cases in other Chinese cities^2,13^. Inter-district flow is a greater promoter of COVID-19 transmission from outbreak locales to other locations than is intra-district flow.

In addition, the relationship between district inflow and the number of confirmed COVID-19 cases before and after alert implementation may affected by several possible reasons such as NPIs and public awareness due to districts with more COVID-19 cases. The implementation of NPIs such as school closures and remote working was expected to reduce COVID-19 transmission due to a reduction in human mobility. Furthermore, public awareness and NPIs use likely reduced the risk of infection during movement^42^. This suggests that the variability in the number of confirmed COVID-19 cases among locations after control measure implementation is better explained by factors such as the public health response than by human mobility^3^.

The finding that older adults’ mobility was significantly related to COVID-19 transmission may be explained by the following reasons. First, because older adults face more health risks related to COVID-19 infection, they likely contribute to large numbers of confirmed cases^34^. Second, older adults may reduce their mobility less than do younger generations when outbreaks occur. In the absence of control measures, most members of younger generations leave their homes for work or school daily, whereas most older adults are retired and less mobile^22^. Regions with larger proportions of the population ranging from 24 to 59 years received the largest effects in mobility caused by the lockdown^19^. Moreover, members of younger generations use social media, where outbreak dangers are emphasized, more than do older adults^22^. Thus, younger generations’ mobility likely decreases obviously, whereas older adults’ mobility is not affected apparently when outbreaks occur. This inference is consistent with Liu et al.’s (2021) reporting that the presence of larger percentages of people aged > 60 years in Chinese cities was related to less mobility reduction after control measure implementation^10^.

The results of this study indicate that the district-level age structure (PC2), household income, and popularity of modern equipment (PC3) were related significantly to the number of confirmed COVID-19 cases before and after alert implementation. The PC2 results indicate that the district-level presence of larger percentages of older adults and fewer members of younger generations was related to a larger number of confirmed COVID-19 cases. This result is consistent with the finding of Mogi and Spijker (2021)^34^, which inferred that the high rate of COVID-19 diagnosis in Italy was likely due to the country’s aged population. The PC3 results indicate that the district-level presence of larger percentages of middle-low-income families and less cable television popularity was related to a larger number of confirmed COVID-19 cases. This result may reflect in part the emphasis on outbreak danger and awareness in the media to reduce exposure and comply with movement restrictions^19,22,43^. On the other hand, socioeconomically disadvantaged segments of the population may be more likely to be exposed to infection due to their social contexts, which could influence infectious disease occurrence^44^. This is consistent with the finding of Waku et al. (2022)^45^, which concluded that death was negatively correlated with the signs of wealth. For instance, low-income status may be related to crowded living conditions, which have been related in turn to a greater risk of infection. People who have lower-income jobs may still need to go to workplaces, whereas people who have higher-income jobs could often work remotely^19^. Furthermore, population segments with lower income levels may not be able to obtain timely high-quality care and nutrition to combat the pandemic^25,46^.

This study has several limitations. First, it was conducted with data from the Taipei metropolitan area, and the results may not be generalizable to other locations, especially rural areas, which have different demographic and regional characteristics. For instance, due to the small amount of district inflow to rural areas, the number of confirmed COVID-19 cases may not be proportional to the amount of human mobility. Thus, the relationships between human mobility and the number of confirmed COVID-19 cases in rural areas need to be clarified. Second, onset dates, rather than reporting dates, were used to determine the daily numbers of new confirmed COVID-19 cases in this study. This approach prevents the problem of delayed reporting, which may influence model results, but the onset dates were self-reported and thus may not be entirely accurate. Third, the current study only investigated the difference in the relationships between population flows and COVID-19 transmission in age groups before and after control measures implementation. However, the changes in the relationships between population flows and COVID-19 transmission were influenced by several possible reasons^47^. The possible reasons influencing the difference in the relationships between population flows and COVID-19 transmission before and after control measures implementation still need to be examined clearly. Fourth, because of the restriction of data availability, the inter-district flows analyzed in this study do not include inflows from districts outside the study area. However, not only inflows from other districts within the study area but also inflows from those outside the study area are better to be included.

Based on the results of this study, we offer four policy suggestions. First, travel (and especially inter-regional travel) restrictions should be implemented as soon as possible in the initial stages of outbreaks of emerging infectious diseases. Second, the rapid implementation of NPIs, such as face mask use and social distancing, contributed to the reduction of COVID-19 transmission caused by human mobility and is recommended in future outbreak situations. Third, the government should strengthen public awareness of epidemic prevention for older adults and provide an economic relief package to help those who have lost their jobs due to a pandemic, especially middle-low-income families. Fourth, the government should consider regional economic, societal, and cultural heterogeneity when formulating local public health responses to a pandemic.

Future research could further explore regional changes in intra-regional movement during COVID-19 outbreaks. In this study, the relationship between intra-district flow and the number of confirmed COVID-19 cases persisted after the implementation of control measures, unlike that of inflow movement. This may be due to people’s maintenance of daily activities and work-related movement in their neighborhoods after alert implementation^19^. These results suggest that the existing epidemic prevention policies less control the intra-regional daily movement during a pandemic. A more complete understanding of local mobility behaviors would aid the formulation of public health responses to community transmission.

## Conclusion

To conclude, this study showed that intra- and inter-regional mobility in age-specific groups had different relationships with COVID-19 transmission before and after the implementation of control measures. The inter-regional mobility was related more strongly to COVID-19 transmission than intra-regional mobility. Furthermore, both the inter-regional mobility of people aged 15–59 and ≥60 years were significantly related to COVID-19 transmission. These findings can aid governments’ formulation of more specific epidemic prevention policies to reduce losses during the COVID-19 pandemic.

### Supplementary Information

Supplementary Tables.

## Data Availability

R code is available at the GitHub repository: https://github.com/JWMHub/Mobility. The datasets of the numbers of confirmed COVID-19 cases and socioeconomic factors analyzed during this study are available from the government’s open data platforms, https://data.gov.tw/. The data on anonymized and aggregated human mobility retrieved from mobile phone data are available from Far EasTone Telecommunications but restrictions apply to the availability of these data, which were used under license for the current study, and so are not publicly available.
