# Supplementary material for: Interregional mobility in different age groups is associated with COVID-19 transmission in the Taipei metropolitan area, Taiwan
Source: Sci Rep. 2023 Oct 12;13:17285. doi: 10.1038/s41598-023-44474-z (PMC10570333; doi:10.1038/s41598-023-44474-z)
Supplement: Supplementary file 1 — Supplementary Tables. [file 41598_2023_44474_MOESM1_ESM.docx]

**Interregional mobility in different age groups is associated with COVID‑19 transmission in the Taipei metropolitan area, Taiwan**

Wei‑Ming Jiang^1^, Tzai‑Hung Wen^2^, Ying-Chi Huang^3^, Hung-Yi Chiou^1,4,5^, Wei J. Chen^6,7^, Chao A. Hsiung^1^, Huey-Kang Sytwu^3^, Hsiao‑Hui Tsou^1,8*^

^1^ Institute of Population Health Sciences, National Health Research Institutes, 35 Keyan Road, Zhunan, Miaoli County 350, Taiwan.

^2^ Department of Geography, National Taiwan University, Taipei, Taiwan.

^3^ National Institute of Infectious Diseases and Vaccinology, National Health Research Institutes, Zhunan, Miaoli County, Taiwan

^4^ School of Public Health, College of Public Health, Taipei Medical University, Taipei, Taiwan

^5^ Master’s Program in Applied Epidemiology, College of Public Health, Taipei Medical University, Taipei, Taiwan

^6^ Center for Neuropsychiatric Research, National Health Research Institutes, Zhunan, Miaoli County, Taiwan

^7^ Institute of Epidemiology and Preventive Medicine, College of Public Health, National Taiwan University, Taipei, Taiwan

^8^ Graduate Institute of Biostatistics, College of Public Health, China Medical University, Taichung, Taiwan

* Correspondence to:

Hsiao-Hui Tsou, Ph.D., Institute of Population Health Sciences, National Health Research Institutes, 35 Keyan Road, Zhunan, Miaoli County 350, Taiwan

E-mail: tsouhh@nhri.org.tw

Tel: 886-37-206-166 ext. 36181

**Supplementary Table S1.** Socioeconomic data collected

| Socioeconomic factor | Release date | Unit |
| --- | --- | --- |
| Demographic structure |  |  |
| Population density | May 2021 | Persons/km^2^ |
| Population aged 0-14 years | December 2020 | Percentage |
| Population aged 15-64 years | December 2020 | Percentage |
| Population aged ≥ 65 years | December 2020 | Percentage |
| Sex ratio | March 2021 | Ratio |
| Dependency ratio | March 2021 | Percentage |
| Child dependency ratio | March 2021 | Percentage |
| Elder dependency ratio | March 2021 | Percentage |
| Aging index | March 2021 | Percentage |
| Natural increase rate | Season 2, 2021 | Per mille |
| Social increase rate | Season 2, 2021 | Per mille |
| Crude marriage rate | Season 2, 2021 | Per mille |
| Crude divorce rate | Season 2, 2021 | Per mille |
| Education level |  |  |
| Doctoral degree | December 2020 | Percentage |
| Master’s degree | December 2020 | Percentage |
| Bachelor’s degree | December 2020 | Percentage |
| Associate degree | December 2020 | Percentage |
| Senior high school | December 2020 | Percentage |
| Junior high school | December 2020 | Percentage |
| Elementary school | December 2020 | Percentage |
| Illiterate | December 2020 | Percentage |
| Economic development |  |  |
| Average disposable household income | December 2019 | Per 1,000 NT$ |
| Average household consumption expenditure | December 2019 | Per 1,000 NT$ |
| Average consolidated income | August 2018 | Per 1,000 NT$ |
| Low-income families | December 2020 | Per mille |
| Middle-low‑income families | December 2020 | Per mille |
| Healthcare |  |  |
| Healthcare expenditure | December 2019 | Percentage |
| Average number of persons served/hospital | December 2020 | Number |
| Average number of hospital beds/persons | December 2020 | Number |
| Equipment modernity |  |  |
| Popularity of cable television | December 2019 | Percentage |
| Popularity of computers | December 2019 | Percentage |
| Popularity of the Internet | December 2019 | Percentage |
| Land use |  |  |
| Commercial areas | 2018-2019 | Percentage |
| Industrial areas | 2018-2019 | Percentage |
| Residential areas | 2018-2019 | Percentage |
| Mixed residential areas | 2018-2019 | Percentage |
| Parks and green spaces | 2018-2019 | Percentage |
| Leisure facilities | 2018-2019 | Percentage |
| NT$: New Taiwan dollar. | | |

**Supplementary Table S2.** The variable loadings of the first 3 PCs.

| Socioeconomic factor | PC1 | PC2 | PC3 |
| --- | --- | --- | --- |
| Demographic structure |  |  |  |
| Population density | -0.11 | 0.10 | 0.11 |
| Population aged 0-14 years | -0.11 | -0.34 | 0.15 |
| Population aged 15-64 years | 0.23 | 0.00 | -0.03 |
| Population aged ≥ 65 years | -0.20 | 0.24 | -0.03 |
| Sex ratio | 0.22 | 0.00 | 0.16 |
| Dependency ratio | -0.23 | 0.02 | 0.05 |
| Child dependency ratio | -0.15 | -0.29 | 0.13 |
| Elder dependency ratio | -0.21 | 0.20 | -0.01 |
| Aging index | -0.08 | 0.37 | -0.13 |
| Natural increase rate | 0.04 | -0.39 | -0.01 |
| Social increase rate | 0.20 | -0.15 | -0.07 |
| Crude marriage rate | 0.18 | -0.16 | -0.18 |
| Crude divorce rate | 0.19 | 0.00 | -0.10 |
| Education level |  |  |  |
| Doctoral degree | -0.22 | -0.03 | -0.06 |
| Master’s degree | -0.24 | -0.03 | -0.07 |
| Bachelor’s degree | -0.23 | 0.00 | -0.13 |
| Associate degree | -0.09 | 0.15 | -0.28 |
| Senior high school | 0.23 | 0.09 | 0.04 |
| Junior high school | 0.22 | 0.03 | 0.12 |
| Elementary school | 0.18 | 0.19 | 0.15 |
| Illiterate | 0.18 | 0.12 | 0.05 |
| Economic development |  |  |  |
| Average disposable household income | -0.21 | -0.07 | 0.15 |
| Average household consumption expenditure | -0.22 | -0.04 | 0.13 |
| Average consolidated income | -0.23 | -0.09 | -0.07 |
| Low‑income families | -0.03 | 0.24 | 0.29 |
| Middle-low‑income families | 0.11 | 0.14 | 0.34 |
| Healthcare |  |  |  |
| Healthcare expenditure | 0.10 | 0.15 | -0.23 |
| Average number of persons served/hospital | 0.19 | -0.05 | 0.03 |
| Average number of hospital beds/persons | -0.11 | 0.01 | 0.04 |
| Modern equipment |  |  |  |
| Popularity of cable television | -0.01 | 0.01 | -0.32 |
| Popularity of computers | -0.14 | -0.08 | 0.25 |
| Popularity of the Internet | -0.09 | -0.08 | 0.30 |
| Land use |  |  |  |
| Commercial areas | -0.02 | -0.20 | -0.21 |
| Industrial areas | 0.16 | -0.20 | 0.08 |
| Residential areas | -0.01 | 0.00 | -0.29 |
| Mixed residential areas | -0.03 | -0.03 | -0.06 |
| Parks and green spaces | 0.00 | 0.03 | 0.02 |
| Leisure facilities | 0.02 | -0.24 | -0.14 |

**Supplementary Table S3.** Regression associations between mobility patterns and the number of confirmed COVID‑19 cases.

|  |  | B1 period | | A1 period | | B2 period | | A2 period | | B3 period | | A3 period | |
| --- | --- | --- | --- | --- | --- | --- | --- | --- | --- | --- | --- | --- | --- |
|  |  | Coefficient  (std. error) | *p* | Coefficient  (std. error) | *p* | Coefficient  (std. error) | *p* | Coefficient  (std. error) | *p* | Coefficient  (std. error) | *p* | Coefficient  (std. error) | *p* |
| a. | Intercept | 2.18 (0.22) | <0.001*** | 4.48 (0.16) | <0.001*** | 3.28 (0.18) | <0.001*** | 4.41 (0.15) | <0.001*** | 4.16 (0.18) | <0.001*** | 4.28 (0.15) | <0.001*** |
|  | Intraflow + Interflow | 0.60 (0.22) | 0.006** | 0.44 (0.16) | 0.004** | 0.59 (0.18) | 0.001** | 0.45 (0.15) | 0.003** | 0.52 (0.18) | 0.003** | 0.42 (0.15) | 0.005** |
|  | Pseudo *r*^2^ | 0.17 | | 0.24 | | 0.24 | | 0.25 | | 0.21 | | 0.21 | |
| b. | Intercept | 2.28 (0.23) | <0.001*** | 4.55 (0.17) | <0.001*** | 3.39 (0.19) | <0.001*** | 4.49 (0.16) | <0.001*** | 4.25 (0.19) | <0.001*** | 4.35 (0.16) | <0.001*** |
|  | Intraflow | 0.24 (0.23) | 0.31 | 0.26 (0.17) | 0.12 | 0.23 (0.20) | 0.24 | 0.24 (0.16) | 0.14 | 0.20 (0.19) | 0.29 | 0.21 (0.16) | 0.21 |
|  | Pseudo *r*^2^ | 0.04 | | 0.08 | | 0.06 | | 0.07 | | 0.05 | | 0.05 | |
| c. | Intercept | 2.12 (0.21) | <0.001*** | 4.44 (0.15) | <0.001*** | 3.22 (0.17) | <0.001*** | 4.36 (0.14) | <0.001*** | 4.10 (0.17) | <0.001*** | 4.23 (0.14) | <0.001*** |
|  | Interflow | 0.81 (0.22) | <0.001*** | 0.59 (0.15) | <0.001*** | 0.81 (0.17) | <0.001*** | 0.61 (0.14) | <0.001*** | 0.78 (0.17) | <0.001*** | 0.59 (0.14) | <0.001*** |
|  | Pseudo *r*^2^ | 0.24 | | 0.33 | | 0.33 | | 0.37 | | 0.32 | | 0.33 | |
| d. | Intercept | 2.11 (0.21) | <0.001*** | 4.43 (0.15) | <0.001*** | 3.21 (0.17) | <0.001*** | 4.36 (0.14) | <0.001*** | 4.08 (0.17) | <0.001*** | 4.23 (0.14) | <0.001*** |
|  | Intraflow | -0.16 (0.25) | 0.52 | -0.09 (0.17) | 0.57 | -0.15 (0.20) | 0.44 | -0.12 (0.16) | 0.43 | -0.22 (0.19) | 0.27 | -0.13 (0.16) | 0.40 |
|  | Interflow | 0.92 (0.25) | <0.001*** | 0.65 (0.17) | <0.001*** | 0.92 (0.20) | <0.001*** | 0.69 (0.16) | <0.001*** | 0.95 (0.19) | <0.001*** | 0.66 (0.16) | <0.001*** |
|  | Pseudo *r*^2^ | 0.25 | | 0.33 | | 0.35 | | 0.38 | | 0.35 | | 0.35 | |

***p* < 0.01, ****p* < 0.001.

**Supplementary Table S4.** Comparing coefficients of the sum of intra‑district flow and inflow before and after the COVID‑19 alert.

|  | B1 period vs. A1 period | | B2 period vs. A2 period | | B3 period vs. A3 period | |
| --- | --- | --- | --- | --- | --- | --- |
|  | Coefficient  (std. error) | *p* | Coefficient  (std. error) | *p* | Coefficient  (std. error) | *p* |
| Intercept | 2.18 (0.19) | <0.001*** | 3.28 (0.16) | <0.001*** | 4.16 (0.17) | <0.001*** |
| Intraflow + Interflow | 0.59 (0.19) | 0.002** | 0.59 (0.17) | <0.001*** | 0.52 (0.17) | 0.002** |
| Period※ | 2.30 (0.26) | <0.001*** | 1.13 (0.23) | <0.001*** | 0.12 (0.23) | 0.61 |
| (Intraflow + Interflow) × Period | -0.15 (0.26) | 0.57 | -0.14 (0.23) | 0.55 | -0.10 (0.24) | 0.68 |
| ***p* < 0.01, ****p* < 0.001. ※Period is a dummy variable that is coded 0 for the period before the COVID‑19 alert and 1 for the period after the COVID‑19 alert. | | | | | | |

**Supplementary Table S5.** Comparing coefficients of interflow before and after the COVID‑19 alert.

|  | B1 period vs. A1 period | | B2 period vs. A2 period | | B3 period vs. A3 period | |
| --- | --- | --- | --- | --- | --- | --- |
|  | Coefficient  (std. error) | *p* | Coefficient  (std. error) | *p* | Coefficient  (std. error) | *p* |
| Intercept | 2.13 (0.18) | <0.001*** | 3.23 (0.16) | <0.001*** | 4.10 (0.16) | <0.001*** |
| Interflow | 0.79 (0.19) | <0.001*** | 0.81 (0.16) | <0.001*** | 0.78 (0.16) | <0.001*** |
| Period※ | 2.31 (0.25) | <0.001*** | 1.14 (0.22) | <0.001*** | 0.14 (0.22) | 0.53 |
| Interflow × Period | -0.20 (0.26) | 0.45 | -0.19 (0.22) | 0.38 | -0.19 (0.22) | 0.39 |
| ***p* < 0.01, ****p* < 0.001. ※Period is a dummy variable that is coded 0 for the period before the COVID‑19 alert and 1 for the period after the COVID‑19 alert. | | | | | | |

**Supplementary Table S6.** Collinearity diagnostics between intra‑ and inter-district flows in the six periods.

|  | Pearson correlation | | Variance inflation factor (VIF) | |
| --- | --- | --- | --- | --- |
|  | *r* | *p* | Interflow | Intraflow |
| B1 period | 0.50 | 0.005 | 1.37 | 1.37 |
| B2 period | 0.50 | 0.005 | 1.37 | 1.36 |
| B3 period | 0.50 | 0.006 | 1.35 | 1.35 |
| A1 period | 0.45 | 0.013 | 1.27 | 1.27 |
| A2 period | 0.45 | 0.014 | 1.27 | 1.27 |
| A3 period | 0.46 | 0.013 | 1.27 | 1.27 |

**Supplementary Table S7**. Regression associations among mobility patterns, PCs, and the number of confirmed COVID‑19 cases in B2, 3 and A2, 3 periods.

|  |  | B2 period | | A2 period | | B3 period | | A3 period | |
| --- | --- | --- | --- | --- | --- | --- | --- | --- | --- |
|  |  | Coefficient  (std. error) | *p* | Coefficient  (std. error) | *p* | Coefficient  (std. error) | *p* | Coefficient  (std. error) | *p* |
| a. | Intercept | 2.93 (0.10) | <0.001*** | 4.23 (0.10) | <0.001*** | 3.77 (0.10) | <0.001*** | 4.12 (0.11) | <0.001*** |
|  | Intraflow + Interflow | 0.83 (0.12) | <0.001*** | 0.68 (0.12) | <0.001*** | 0.81 (0.12) | <0.001*** | 0.66 (0.12) | <0.001*** |
|  | PC1 | 0.05 (0.03) | 0.09 | -0.03 (0.03) | 0.23 | 0.04 (0.03) | 0.17 | -0.04 (0.03) | 0.15 |
|  | PC2 | 0.23 (0.05) | <0.001*** | 0.13 (0.05) | 0.007** | 0.22 (0.05) | <0.001*** | 0.11 (0.05) | 0.02* |
|  | PC3 | 0.20 (0.06) | <0.001*** | 0.19 (0.06) | 0.001** | 0.23 (0.05) | <0.001*** | 0.19 (0.06) | 0.001** |
|  | Pseudo *r*^2^ | 0.76 | | 0.66 | | 0.80 | | 0.61 | |
| b. | Intercept | 2.97 (0.11) | <0.001*** | 4.28 (0.12) | <0.001*** | 3.81 (0.11) | <0.001*** | 4.16 (0.12) | <0.001*** |
|  | Intraflow | 0.78 (0.13) | <0.001*** | 0.73 (0.15) | <0.001*** | 0.79 (0.13) | <0.001*** | 0.72 (0.15) | <0.001*** |
|  | PC1 | -0.04 (0.03) | 0.14 | -0.09 (0.03) | 0.004** | -0.05 (0.03) | 0.06 | -0.10 (0.03) | 0.002** |
|  | PC2 | 0.25 (0.05) | <0.001*** | 0.15 (0.05) | 0.004** | 0.24 (0.05) | <0.001*** | 0.13 (0.05) | 0.01* |
|  | PC3 | 0.31 (0.07) | <0.001*** | 0.24 (0.07) | 0.001** | 0.32 (0.07) | <0.001*** | 0.25 (0.08) | 0.001** |
|  | Pseudo *r*^2^ | 0.73 | | 0.55 | | 0.75 | | 0.51 | |
| c. | Intercept | 2.96 (0.11) | <0.001*** | 4.25 (0.11) | <0.001*** | 3.81 (0.11) | <0.001*** | 4.14 (0.11) | <0.001*** |
|  | Interflow | 0.87 (0.15) | <0.001*** | 0.64 (0.12) | <0.001*** | 0.84 (0.14) | <0.001*** | 0.60 (0.13) | <0.001*** |
|  | PC1 | 0.09 (0.04) | 0.02* | 0.01 (0.03) | 0.83 | 0.08 (0.03) | 0.02* | -0.001 (0.03) | 0.98 |
|  | PC2 | 0.22 (0.05) | <0.001*** | 0.12 (0.05) | 0.01* | 0.21 (0.05) | <0.001*** | 0.11 (0.05) | 0.03* |
|  | PC3 | 0.11 (0.06) | 0.06 | 0.10 (0.06) | 0.08 | 0.13 (0.06) | 0.02* | 0.10 (0.06) | 0.09 |
|  | Pseudo *r*^2^ | 0.72 | | 0.62 | | 0.75 | | 0.57 | |
| d. | Intercept | 2.93 (0.10) | <0.001*** | 4.23 (0.10) | <0.001*** | 3.77 (0.10) | <0.001*** | 4.12 (0.11) | <0.001*** |
|  | Intraflow | 0.45 (0.17) | 0.006** | 0.33 (0.16) | 0.04* | 0.45 (0.16) | 0.003** | 0.35 (0.17) | 0.04* |
|  | Interflow | 0.48 (0.19) | 0.01* | 0.46 (0.15) | 0.002** | 0.48 (0.17) | 0.005** | 0.43 (0.16) | 0.006** |
|  | PC1 | 0.03 (0.04) | 0.45 | -0.03 (0.03) | 0.37 | 0.02 (0.04) | 0.56 | -0.04 (0.03) | 0.24 |
|  | PC2 | 0.23 (0.05) | <0.001*** | 0.13 (0.05) | 0.007** | 0.22 (0.05) | <0.001*** | 0.11 (0.05) | 0.02* |
|  | PC3 | 0.24 (0.07) | <0.001*** | 0.18 (0.07) | 0.007** | 0.25 (0.06) | <0.001*** | 0.19 (0.07) | 0.01** |
|  | Pseudo *r*^2^ | 0.77 | | 0.66 | | 0.80 | | 0.61 | |
| **p* < 0.05, ***p* < 0.01, ****p* < 0.001. | | | | | | | | | |

**Supplementary Table S8.** Regression associations among the intra‑district flow of people aged 15-59 and ≥60 years, PCs, and the number of confirmed COVID‑19 cases.

|  |  | B1 period | | A1 period | | B2 period | | A2 period | | B3 period | | A3 period | |
| --- | --- | --- | --- | --- | --- | --- | --- | --- | --- | --- | --- | --- | --- |
|  |  | Coefficient  (std. error) | *p* | Coefficient  (std. error) | *p* | Coefficient  (std. error) | *p* | Coefficient  (std. error) | *p* | Coefficient  (std. error) | *p* | Coefficient  (std. error) | *p* |
| a. | Intercept | 1.83 (0.16) | <0.001*** | 4.31 (0.12) | <0.001*** | 2.96 (0.11) | <0.001*** | 4.27 (0.11) | <0.001*** | 3.80 (0.11) | <0.001*** | 4.15 (0.12) | <0.001*** |
|  | Intraflow  (15-59) | 0.80 (0.18) | <0.001*** | 0.75 (0.15) | <0.001*** | 0.79 (0.13) | <0.001*** | 0.76 (0.15) | <0.001*** | 0.80 (0.13) | <0.001*** | 0.76 (0.15) | <0.001*** |
|  | PC1 | -0.05 (0.04) | 0.24 | -0.09 (0.03) | 0.004** | -0.06 (0.03) | 0.03* | -0.11 (0.03) | <0.001*** | -0.07 (0.03) | 0.01* | -0.12 (0.03) | <0.001*** |
|  | PC2 | 0.27 (0.08) | <0.001*** | 0.18 (0.05) | <0.001*** | 0.26 (0.05) | <0.001*** | 0.17 (0.05) | 0.001** | 0.25 (0.05) | <0.001 | 0.15 (0.05) | 0.006** |
|  | PC3 | 0.31 (0.10) | 0.001** | 0.23 (0.07) | 0.001** | 0.31 (0.07) | <0.001*** | 0.25 (0.07) | <0.001*** | 0.32 (0.06) | <0.001*** | 0.26 (0.07) | <0.001*** |
|  | Pseudo *r*^2^ | 0.58 | | 0.59 | | 0.74 | | 0.58 | | 0.77 | | 0.54 | |
| b. | Intercept | 1.89 (0.17) | <0.001*** | 4.37 (0.13) | <0.001*** | 3.01 (0.13) | <0.001*** | 4.34 (0.13) | <0.001*** | 3.86 (0.12) | <0.001*** | 4.21 (0.13) | <0.001*** |
|  | Intraflow  (≥60) | 0.76 (0.21) | <0.001*** | 0.67 (0.17) | <0.001*** | 0.77 (0.15) | <0.001*** | 0.64 (0.16) | <0.001*** | 0.78 (0.15) | <0.001*** | 0.61 (0.17) | <0.001*** |
|  | PC1 | 0.001 (0.04) | 0.99 | -0.03 (0.03) | 0.40 | -0.01 (0.03) | 0.65 | -0.04 (0.03) | 0.19 | -0.02 (0.03) | 0.50 | -0.05 (0.03) | 0.13 |
|  | PC2 | 0.21 (0.08) | 0.01* | 0.14 (0.06) | 0.02* | 0.20 (0.06) | 0.001** | 0.13 (0.06) | 0.03* | 0.19 (0.06) | <0.001*** | 0.11 (0.06) | 0.06 |
|  | PC3 | 0.30 (0.11) | 0.005** | 0.22 (0.08) | 0.009** | 0.31 (0.08) | <0.001*** | 0.22 (0.08) | 0.007** | 0.32 (0.07) | <0.001*** | 0.22 (0.09) | 0.01* |
|  | Pseudo *r*^2^ | 0.51 | | 0.46 | | 0.66 | | 0.43 | | 0.69 | | 0.39 | |
| c. | Intercept | 2.26 (0.23) | <0.001*** | 4.55 (0.17) | <0.001*** | 3.36 (0.19) | <0.001*** | 4.49 (0.16) | <0.001*** | 4.22 (0.19) | <0.001*** | 4.35 (0.16) | <0.001*** |
|  | Intraflow  (15-59) | -0.36 (0.60) | 0.54 | 0.06 (0.40) | 0.88 | -0.49 (0.50) | 0.32 | 0.08 (0.38) | 0.83 | -0.49 (0.50) | 0.32 | 0.08 (0.38) | 0.83 |
|  | Intraflow  (≥60) | 0.74 (0.59) | 0.21 | 0.22 (0.40) | 0.58 | 0.88 (0.50) | 0.07 | 0.18 (0.38) | 0.65 | 0.86 (0.50) | 0.08 | 0.13 (0.38) | 0.74 |
|  | Pseudo *r*^2^ | 0.07 | | 0.08 | | 0.11 | | 0.07 | | 0.10 | | 0.05 | |
| **p* < 0.05, ***p* < 0.01, ****p* < 0.001. | | | | | | | | | | | | | |

**Supplementary Table S9.** Regression associations among the interflow of people aged 15-59 and ≥60 years, PCs, and the number of confirmed COVID‑19 cases.

|  |  | B1 period | | A1 period | | B2 period | | A2 period | | B3 period | | A3 period | |
| --- | --- | --- | --- | --- | --- | --- | --- | --- | --- | --- | --- | --- | --- |
|  |  | Coefficient  (std. error) | *p* | Coefficient  (std. error) | *p* | Coefficient  (std. error) | *p* | Coefficient  (std. error) | *p* | Coefficient  (std. error) | *p* | Coefficient  (std. error) | *p* |
| a. | Intercept | 1.83 (0.16) | <0.001*** | 4.40 (0.14) | <0.001*** | 2.97 (0.11) | <0.001*** | 4.25 (0.11) | <0.001*** | 3.81 (0.11) | <0.001*** | 4.14 (0.11) | <0.001*** |
|  | Interflow  (15-59) | 0.82 (0.19) | <0.001*** | 0.48 (0.15) | 0.002** | 0.81 (0.14) | <0.001*** | 0.60 (0.12) | <0.001*** | 0.78 (0.13) | <0.001*** | 0.57 (0.12) | <0.001*** |
|  | PC1 | 0.08 (0.05) | 0.09 | 0.005 (0.04) | 0.89 | 0.06 (0.03) | 0.06 | -0.01 (0.03) | 0.61 | 0.05 (0.03) | 0.10 | -0.02 (0.03) | 0.48 |
|  | PC2 | 0.24 (0.08) | 0.002** | 0.03 (0.06) | 0.65 | 0.23 (0.06) | <0.001*** | 0.13 (0.05) | 0.007** | 0.22 (0.05) | <0.001*** | 0.12 (0.05) | 0.05* |
|  | PC3 | 0.12 (0.08) | 0.15 | 0.03 (0.07) | 0.69 | 0.11 (0.06) | 0.07 | 0.09 (0.06) | 0.10 | 0.13 (0.06) | 0.02* | 0.09 (0.06) | 0.11 |
|  | Pseudo *r*^2^ | 0.57 | | 0.41 | | 0.72 | | 0.61 | | 0.75 | | 0.56 | |
| b. | Intercept | 1.84 (0.16) | <0.001*** | 4.35 (0.13) | <0.001*** | 2.98 (0.12) | <0.001*** | 4.26 (0.11) | <0.001*** | 3.82 (0.11) | <0.001*** | 4.14 (0.11) | <0.001*** |
|  | Interflow  (≥60) | 1.05 (0.25) | <0.001*** | 0.66 (0.18) | <0.001*** | 1.01 (0.18) | <0.001*** | 0.74 (0.15) | <0.001*** | 0.99 (0.17) | <0.001*** | 0.70 (0.15) | <0.001*** |
|  | PC1 | 0.16 (0.06) | 0.007** | 0.07 (0.04) | 0.13 | 0.14 (0.04) | 0.002** | 0.05 (0.04) | 0.14 | 0.13 (0.04) | 0.002** | 0.05 (0.04) | 0.22 |
|  | PC2 | 0.16 (0.08) | 0.04* | 0.03 (0.06) | 0.65 | 0.16 (0.06) | 0.006** | 0.09 (0.05) | 0.09 | 0.15 (0.05) | 0.003** | 0.07 (0.05) | 0.16 |
|  | PC3 | 0.12 (0.08) | 0.15 | 0.06 (0.07) | 0.40 | 0.11 (0.06) | 0.06 | 0.11 (0.06) | 0.05 | 0.14 (0.06) | 0.02* | 0.11 (0.06) | 0.06 |
|  | Pseudo *r*^2^ | 0.57 | | 0.50 | | 0.70 | | 0.60 | | 0.74 | | 0.55 | |
| c. | Intercept | 2.08 (0.21) | <0.001*** | 4.42 (0.14) | <0.001*** | 3.19 (0.16) | <0.001*** | 4.35 (0.13) | <0.001*** | 4.04 (0.16) | <0.001*** | 4.22 (0.13) | <0.001*** |
|  | Interflow  (15-59) | 0.05 (0.64) | 0.94 | 0.01 (0.36) | 0.97 | -0.02 (0.52) | 0.98 | 0.05 (0.32) | 0.87 | -0.19 (0.50) | 0.70 | 0.02 (0.32) | 0.94 |
|  | Interflow  (≥60) | 0.83 (0.65) | 0.20 | 0.67 (0.35) | 0.06 | 0.89 (0.52) | 0.09 | 0.64 (0.32) | 0.04* | 1.08 (0.50) | 0.03* | 0.65 (0.32) | 0.04* |
|  | Pseudo *r*^2^ | 0.28 | | 0.36 | | 0.39 | | 0.40 | | 0.42 | | 0.38 | |
| **p* < 0.05, ***p* < 0.01, ****p* < 0.001. | | | | | | | | | | | | | |

**Supplementary Table S10.** Collinearity diagnostics between intra-district flows of people aged 15-59 and ≥60 years in the six periods.

|  | Pearson correlation | | Variance inflation factor (VIF) | |
| --- | --- | --- | --- | --- |
|  | *r* | *p* | Intraflow (15-59) | Intraflow (≥60) |
| B1 period | 0.92 | <0.001 | 6.69 | 6.69 |
| B2 period | 0.92 | <0.001 | 6.74 | 6.74 |
| B3 period | 0.92 | <0.001 | 6.84 | 6.84 |
| A1 period | 0.90 | <0.001 | 5.51 | 5.51 |
| A2 period | 0.90 | <0.001 | 5.48 | 5.48 |
| A3 period | 0.90 | <0.001 | 5.42 | 5.42 |

**Supplementary Table S11.** Collinearity diagnostics between inter-district flows of people aged 15-59 and ≥60 years in the six periods.

|  | Pearson correlation | | Variance inflation factor (VIF) | |
| --- | --- | --- | --- | --- |
|  | *r* | *p* | Interflow (15-59) | Interflow (≥60) |
| B1 period | 0.95 | <0.001 | 9.21 | 9.21 |
| B2 period | 0.95 | <0.001 | 9.65 | 9.65 |
| B3 period | 0.95 | <0.001 | 9.33 | 9.33 |
| A1 period | 0.91 | <0.001 | 5.92 | 5.92 |
| A2 period | 0.91 | <0.001 | 5.49 | 5.49 |
| A3 period | 0.90 | <0.001 | 5.41 | 5.41 |
